# Talin: a protein designed for mechanotransduction

**DOI:** 10.1042/ETLS20180179

**Published:** 2018-12-21

**Authors:** Keith Burridge

**Affiliations:** Department of Cell Biology and Physiology, Lineberger Comprehensive Cancer Center, University of North Carolina, Chapel Hill, NC 27599, U.S.A.

**Keywords:** mechanotransduction, protein, talin

## Abstract

Mechanotransduction, the topic of this volume, has become a major area of cell biological research. That cells respond to their external environments has been known for decades; however, research was largely confined to studying how cells respond to soluble factors and not mechanical forces. Here, I will use talin, a canonical mechanosensitive protein, to illustrate certain emerging concepts.

Talin's location in focal adhesions and its binding to both integrins and actin filaments positions it for bidirectional transmission of tension across the cell membrane, i.e. transmitting forces generated internally or imposed externally on cells [1,2]. The idea that tension generated within stress fibers contributes both to their own assembly and to the assembly of focal adhesions goes back nearly 40 years [3,4]. The original explanation was that mechanical tension, together with myosin-mediated bundling of actin filaments, clusters integrins to nucleate focal adhesion assembly. Indeed, later work showed that direct application of mechanical tension on cells promotes the growth of focal adhesions [5] and the recruitment of proteins such as vinculin to these sites [6]. The possibility that cryptic protein-binding sites could be exposed by tension to promote assembly was not originally considered, but subsequent work has revealed that this is critical for recruitment of proteins to focal adhesions [7]. Tension-mediated unfolding of protein domains was originally analyzed theoretically by Erickson [8], who considered how the giant protein titin can change its length by as much as 400% during muscle contraction and stretching. Calculating the force needed to reversibly unfold the tandem fibronectin type III repeats and immunoglobulin domains in titin to generate the stretched length, Erickson found that this force was within the range generated by myosin motors. He extended the analysis to fibronectin and suggested that mechanical force-driven unfolding could also occur in other proteins containing similar tandem repeat sequences. It was subsequently shown that tension promotes fibronectin assembly [9] and exposes cryptic sites [10].

Talin also contains tandem repeat sequences within its rod domain and one of its unexpected features is that it contains 11 vinculin-binding sites, with most being cryptic within the purified protein [11]. Simulated molecular dynamics analysis revealed that these potentially could be exposed by mechanical tension leading to vinculin recruitment [12,13]. Subsequent studies using single molecule atomic force microscopy revealed that, indeed, these vinculin-binding sites are exposed when talin experiences physiologically relevant mechanical tension [7,14].

In addition to recruiting additional components to focal adhesions, the stretching of talin has been implicated in signal transduction. For example, calpain-mediated cleavage of talin, usually considered in the context of disassembly [15], is promoted by tension and occurs during adhesion assembly [16]. Expression of non-cleavable talin inhibited not only adhesion maturation but, unexpectedly, also cell growth. This was rescued by co-expression of the talin rod [16]. It will be exciting to learn the signaling pathways involved. Tension on talin also affects the Rho GTPase signaling pathway, which regulates contractility and focal adhesion assembly. Deleted in liver cancer 1 (DLC1) is a RhoGAP and negative regulator of RhoA signaling that binds the unstretched form of talin [17,18]. It is released in response to tension [19]. Recent studies show that when bound to talin, DLC1 is active and antagonizes RhoA activity, decreasing myosin-driven contractility [19]. At first glance, this appears counter-intuitive because this would lead to positive rather than negative feedback, i.e. release of DLC1 by stretching talin would remove this inhibitory signal and further increase contractility. However, a more complex regulatory scheme has been proposed. Tension on small adhesions is often greater than on large adhesions [2,20]. The initial positive feedback increasing contractility and tension on talin will promote growth, but as adhesions grow the load will be carried by more components, thereby decreasing the level of tension experienced by any single talin molecule. This is predicted to favor refolding of the talin domain to which DLC1 binds, consequently activating DLC1 to inhibit RhoA and decrease contractility [19]. An alternative explanation is that talin can bind actin at multiple sites along the molecule, either directly or indirectly via vinculin. One can imagine a scenario where the initial tension releases DLC1 and recruits vinculin to some of the vinculin-binding sites. These may provide a different vector of force on talin such that the domain responsible for binding DLC1 is no longer under high tension, allowing it to refold, bind, and activate DLC1.

The stretching of talin illustrates how mechanical force can affect a protein's interactions to affect cytoskeletal organization and cell signaling. This is discussed more thoroughly elsewhere [21], but many thought-provoking questions remain to be answered. As more talin-binding partners are identified, it will be important to learn how tension on talin affects these interactions. Also, because talin has multiple connections to actin, it will be interesting to determine if there are situations where tension can be directed so as to stretch specific domains in talin to selectively affect particular signaling pathways.

## Abbreviations

DLC1: deleted in liver cancer 1
